# Chikungunya Virus Infection of Cell Lines: Analysis of the East, Central and South African Lineage

**DOI:** 10.1371/journal.pone.0031102

**Published:** 2012-01-27

**Authors:** Nitwara Wikan, Prirayapak Sakoonwatanyoo, Sukathida Ubol, Sutee Yoksan, Duncan R. Smith

**Affiliations:** 1 Institute of Molecular Biosciences, Mahidol University, Bangkok, Thailand; 2 Department of Clinical Pathology, Faculty of Medicine, Vajira Hospital, Navamindradhiraj University, Bangkok, Thailand; 3 Department of Microbiology, Faculty of Science, Mahidol University, Bangkok, Thailand; 4 Center for Emerging and Neglected Infectious Diseases, Mahidol University, Bangkok, Thailand; Agency for Science, Technology and Research - Singapore Immunology Network, Singapore

## Abstract

Chikungunya virus (CHIKV) is a re-emerging mosquito borne alphavirus that has caused large scale epidemics in the countries around the Indian Ocean, as well as leading to autochthonous transmission in some European countries. The transmission of the disease has been driven by the emergence of an African lineage of CHIKV with enhanced transmission and dissemination in *Aedes* mosquito hosts. Two main genotypes of this lineage have been circulating, characterized by the presence of a substitution of a valine for an alanine at position 226 of the E1 protein. The outbreak, numbering in millions of cases in the infected areas, has been associated with increasing numbers of cases with non-classical presentation including encephalitis and meningitis. This study sought to compare the original Ross strain with two isolates from the recent outbreak of chikungunya fever in respect of infectivity and the induction of apoptosis in eight mammalian cell lines and two insect cell lines, in addition to generating a comprehensive virus production profile for one of the newer isolates. Results showed that in mammalian cells there were few differences in either tropism or pathogenicity as assessed by induction of apoptosis with the exception of Hela cells were the recent valine isolate showed less infectivity. The Aedes albopictus C6/36 cell line was however significantly more permissive for both of the more recent isolates than the Ross strain. The results suggest that the increased infectivity seen in insect cells derives from an evolution of the CHIKV genome not solely associated with the E1:226 substitution.

## Introduction

Chikungunya fever is a mosquito transmitted viral disease that is classically characterized by fever, rash and a severe, often persistent, joint pain (arthralgia) and while rarely fatal, widespread outbreaks of chikungunya fever have led to significant long term morbidity and economic loss [1], [2], [3]. Chikungunya fever was first formally described in 1955 following an outbreak of the disease in southern Tanzania in 1952 [4] and the disease occurs in Africa, the Indian subcontinent and Southeast Asia [5]. The severe arthralgia can serve to discriminate the disease from the largely similar dengue fever [6] and while the pain is normally resolved in a few days or weeks, prolonged joint pain can persist for several months or even years [7], [8]. In the last few decades, there have been an increasing number of reports of severe complications of chikungunya fever. In the 1963 outbreak in India, neurological and haematological complications were reported for the first time [9], [10] and recent outbreaks of the disease have been associated with periventricular encephalitis and meningoencephalitis in neonates [11], [12] as well as renal and hepatic dysfunction [13] ophthalmic involvement, hypokalemic paralysis, hearing loss, Guillain Barre Syndrome and acute flaccid paralysis [14], [15], [16], [17], [18], [19], [20], [21] and occasional deaths have been reported [22], [23], [24], [25]. More recently, neurologic involvement has been reported in Thailand [26], while deaths as a result of chikungunya infection have been reported in Malaysia [24], [27].

The causative agent of chikungunya fever, the Chikungunya virus (CHIKV), is an *Alphavirus* belonging to the family *Togaviridae* and was originally isolated from the southern Tanzanian outbreak in 1952 [28]. The genome consists of a positive sense single stranded RNA molecule of approximately 11.8 kb which has both a 5′-methylguanylate cap and a 3′-polyadenylate tail [29] and encodes for four non-structural proteins (nsP1 to nsP4), three structural proteins (C, E1 and E2) and two small peptides (E3 and 6 K) in two open reading frames [29], [30].

Sequence analysis of genomes isolated from different geographic areas has shown that there are three distinct clades of CHIKV, which are the so called West African, East, Central and South African (ECSA) and Asian lineages, with Asian lineage arising from the ECSA lineage some 50 to 300 years ago [31], [32]. In Africa, CHIKV is believed to be maintained in a primarily sylvatic cycle involving wild, non human primates and forest dwelling *Aedes* mosquitoes such as *Aedes furcifer* and *Aedes taylori*, while in Asia the virus is believed to be transmitted primarily in a mosquito-human-mosquito cycle during epidemics with the urban *Aedes aegypti* being the primary transmission vector [13], [33].

In 2005/2006 significant attention was focused on CHIKV as a consequence of a massive outbreak of chikungunya fever on the Indian Ocean island of La Reunion that eventually affected nearly 40% of the population [34]. Subsequent outbreaks of chikungunya fever in India, Sri Lanka, Malaysia, Singapore and Thailand in the following years resulted in millions of infected patients and the large numbers of patients coupled with the absence of a vaccine and lack of a specific treatment made chikungunya fever a significant worldwide public health problem [33], [35], [36]. Sequence analysis showed that the CHIKV responsible for the outbreak belonged to the ECSA clade of CHIKV [37], [38], [39], [40], [41]. More importantly, much of the outbreak was being driven by adaptation of the virus to *Aedes albopictus* mosquitoes as opposed to the classical vector of *Aedes aegypti* and the adaptation of the virus to *Aedes albopictus* was coupled with the emergence of CHIKV bearing a hallmark substitution in the E1 protein at position 226 [40]. This change, the substitution of a valine for an alanine (E1: A226V) increased the transmissibility and dissemination of the virus in *Aedes albopictus* as well as possibly in *Aedes aegypti* mosquitoes [42]. Surprisingly, evidence suggests that this substitution has arisen independently at least three times during the last few years in what is believed to be a rare example of convergent evolution [33], [38], [43]. While the mechanism by which this substitution increases the fitness of the virus in *Aedes albopictus* mosquitoes remains poorly understood [42], [44], the consequences are significant. An autochthonous outbreak of chikungunya fever in Italy in 2007 resulted from convergence of high levels of *Aedes albopictus* in Italy at the time of a visit of an asymptomatic CHIKV infected person [45], [46], raising the possibility of much wider dissemination of CHIKV as a result of its adaptation to the *Aedes albopictus* mosquito [47].

At this point there is little evidence how or if the E1: A226V substitution alters the interaction with mammalian cells or whether the currently circulating ECSA lineages (E1: A226 or E1: 226V) differ significantly from the original ECSA lineage isolated in Africa in the early 1950 s. To start to address this question, we examined the ability of three ECSA lineages to infect a number of mammalian and insect cell lines (both *Aedes aegypti* and *Aedes albopictus*), and determined the consequences of infection by determining whether apoptosis was induced. Two of the CHIKVs were recent isolates, while the third CHIKV was the prototype Ross strain.

The tissue tropism of CHIKV in humans remains to be fully defined, but previous studies have shown that a number of cell types including epithelial, endothelial and fibroblast cells are all susceptible to infection [25]. Monocyte derived macrophages are susceptible to infection and are believed to play a critical role in the disease pathology [48], while monocytes and monocytic cells lines are variably susceptible [25], [49]. Consistent with the reports of neurological involvement, both neurons and glial cells are believed to be susceptible to infection [11]. In a non-human primate model, persistent infection of liver tissues, as well as significant levels of hepatocyte cell death implicates the involvement of hepatocytes in the disease [48]. We therefore selected a range of cell lines including CHME-5 (human embryonic fetal microglial), HEK293T/17 (human embryonic kidney), HepG2 (human hepatocarcinoma), MRC-5 (human lung fibroblast), Hela (human cervical epithelial), SW-982 (human synovial sarcoma), U937 (human histiocytic lymphoma) and Vero (African Green Monkey Kidney) cells and assessed these cells for infection and induction of apoptosis in response to infection. In mammalian cells surprisingly few differences were found between the different isolates while insect cells showed the largest difference between Ross strain and the later isolates in C6/36 Aedes albopictus cells.

## Results

Three ECSA CHIKV isolates were used in this study. The first was an ECSA E1: 226 V isolate obtained from a patient in Thailand in 2009 and passaged twice in *Aedes albopictus* C6/36 cells before further passage in Vero cells. The second was an ECSA E1: A226 isolate (a kind gift of the Emerging Infectious Disease Program, Duke-NUS Medical School, Singapore) which was initially passaged in BHK-21 and twice further in Vero. The third isolate was ECSA Ross strain, which was passaged 5 times through Vero (and which is also E1: A226). The identity of all isolates was confirmed by DNA sequencing (data not shown). Virus titer for each isolate was determined by standard plaque assay on Vero cells. For the ECSA E1: 226 V isolate, virus production from 8 mammalian cell lines (CHME-5, HEK293T/17, HepG2, MRC-5, Hela, SW-982, U937 and Vero) and 2 insect cell lines, the *Aedes albopictus* derived C6/36 cell line [50] and the *Aedes aegypti* derived CCL-125 cell line [50] was determined by standard plaque assay on Vero cells after infection at a multiplicity of infection (m.o.i.) of 1. All three isolates were then used to infect the same 10 cell lines at a multiplicity of infection (m.o.i.) of 1 and the susceptibility of each cell line to the virus was quantified by flow cytometry using an anti-alphavirus monoclonal antibody in parallel with determination of the induction of apoptosis also by flow cytometry after staining cells with FITC-conjugated Annexin V and propidium iodide. All experiments were undertaken independently in triplicate. For selected cell lines immunocytochemistry was used to directly visualize infection.

### The ECSA E1: 226 V isolate

We initially determined the profile of virus productivity for the ECSA E1: 226 V isolate after infection of CHME-5, HEK293T/17, HepG2, MRC-5, Hela, SW-982, U937, Vero and C6/36. Titer of virus in the supernatant was determined by standard plaque assay immediately upon addition of the virus to the cells at m.o.i. 1 (−2 h.p.i.) and at 4, 8 and 12 h.p.i. as well as on 1, 3 and 5 days p.i. (d.p.i.). Some cell lines also included a 0 hour and a 2 d.p.i. time point. Each experiment was undertaken independently in triplicate.

**Figure 1:**
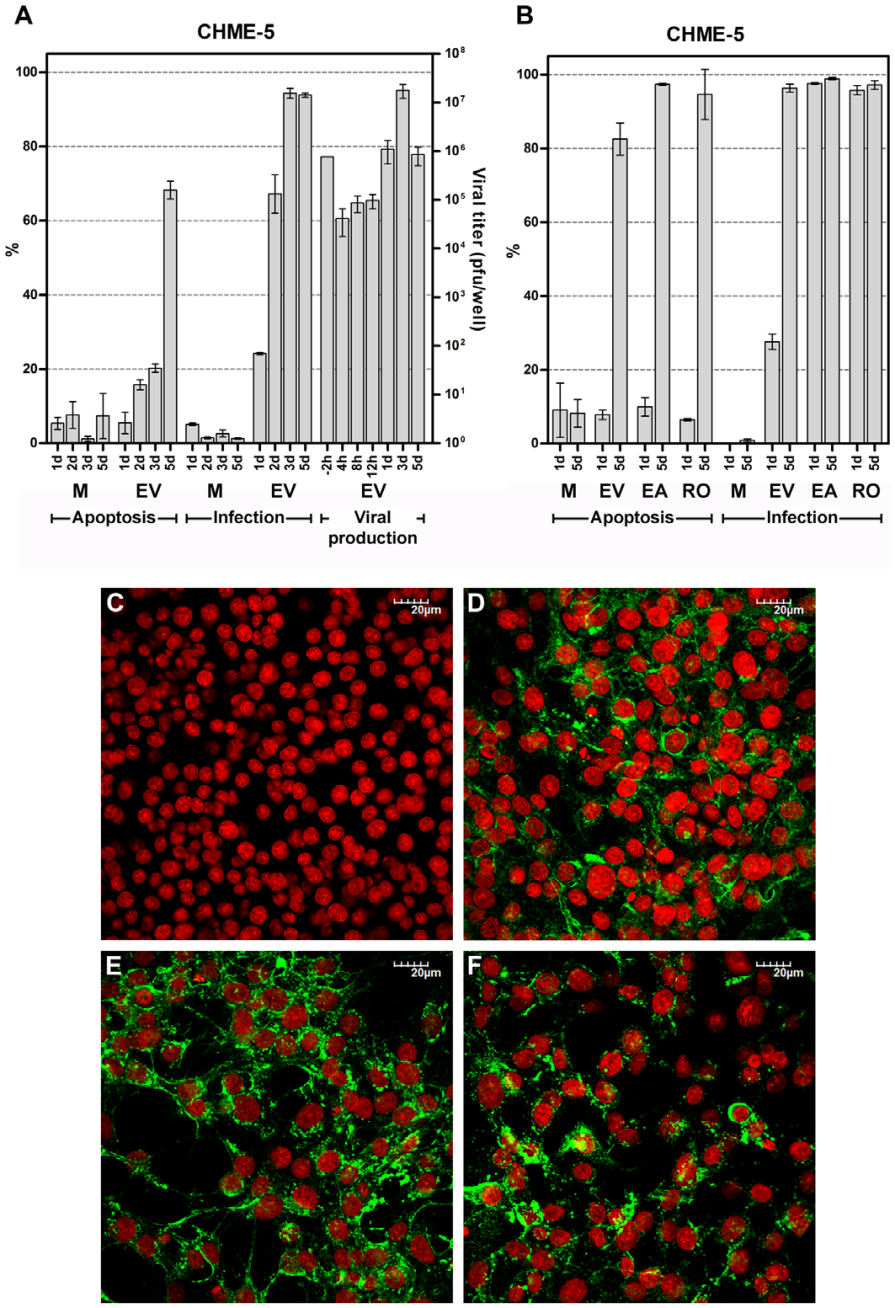
CHIKV infection of CHME-5 human microglial cells. A. CHME-5 cells were mock infected (M) or infected with CHIKV ECSA E1: 226 V (EV) at m.o.i. of 1. Infected cells and culture medium were collected daily and the medium assayed for levels of infectious CHIKV at the times indicated by standard plaque assay on Vero cells, while cells were assayed for infectivity and induction of apoptosis by flow cytometry after staining with an anti-alphavirus monoclonal antibody and FITC-conjugated Annexin V/propidium iodide respectively. B. CHME-5 cells were mock infected (M) or infected with ECSA CHIKV E1: A226 (EA), ECSA CHIKV E1: 226 V (EV) or Ross strain (RO) at m.o.i. of 1 and assayed for infectivity and induction of apoptosis by flow cytometry on the days indicated. All experiments were undertaken independently in triplicate with duplicate analysis of virus titers. Error bars show S.D. C to F. CHME-5 cells were mock infected (C) or infected with ECSA CHIKV E1: 226 V (D), ECSA CHIKV E1: A226 (E) or Ross strain (F) and on day 2 p.i. stained with a mouse anti-alphavirus monoclonal antibody followed by a FITC conjugated goat anti-mouse IgG polyclonal antibody (green). Nuclei of cells were stained with TO-PRO-3 iodide (red). Non-contrast adjusted merged images are shown.

**Figure 2:**
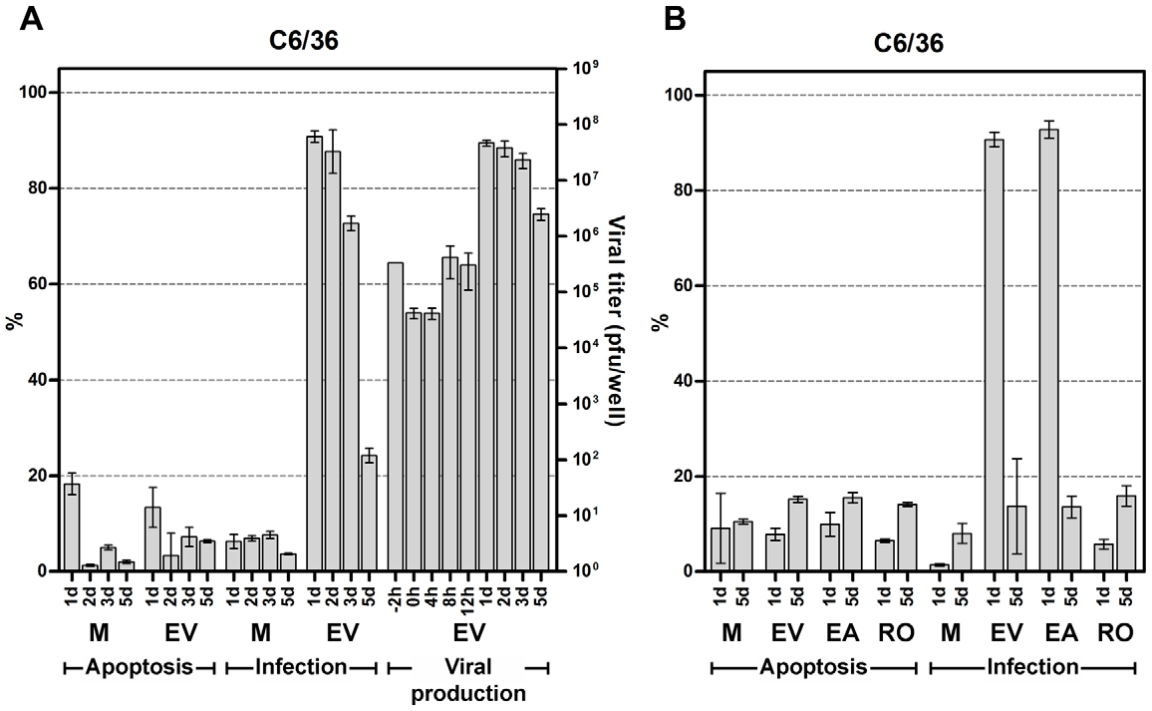
CHIKV infection of C6/36 *Aedes albopictus* cells. A. C6/36 cells were mock infected (M) or infected with CHIKV ECSA E1: 226 V (EV) at m.o.i. of 1. Infected cells and culture medium were collected daily and the medium assayed for levels of infectious CHIKV at the times indicated by standard plaque assay on Vero cells, while cells were assayed for infectivity and induction of apoptosis by flow cytometry after staining with an anti-alphavirus monoclonal antibody and FITC-conjugated Annexin V/propidium iodide respectively. B. C6/36 cells were mock infected (M) or infected with ECSA CHIKV E1: A226 (EA), ECSA CHIKV E1: 226 V (EV) or Ross strain (RO) at m.o.i. of 1 and assayed for infectivity and induction of apoptosis by flow cytometry on the days indicated. All experiments were undertaken independently in triplicate with duplicate analysis of virus titers. Error bars show S.D.

Results of the plaque assays for CHME-5 (Figure 1A), HepG2 (Figure S1A) Hela (Figure S2A) HEK293T (Figure S3A), Vero (Figure S4A), SW-982 (Figure S5A), MRC-5 (Figure S6A) and C6/36 (Figure 2A) showed that in the first 12 h post infection there was a decrease of virus levels in the medium, corresponding to virus binding and/or internalization, but by 1 d.p.i. (C6/36, Vero and MRC-5), 2 d.p.i. (HEK293T/17) or 3 d.p.i. (Hela, CHME-5 and HepG2) *de novo* virus production over the level of input virus had clearly occurred after which titer generally started to decline. SW-982 (Figure S5A) showed a characteristic drop in virus titer following the infection period however there was only a slight increase in virus titer on day 2 p.i. which did not reach input levels, followed by a rapid decline of titer on days 3 to 5 p.i. U937 infections showed no decrease of virus titer in the first 12 hours p.i., and the titer declined over the next 5 days to 2Log lower than the input level (Figure S7A).

**Figure 3:**
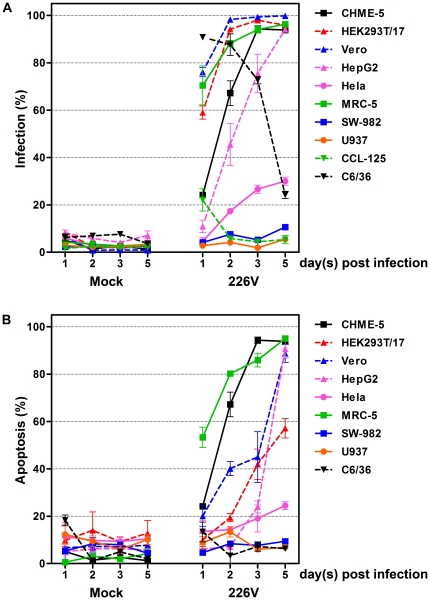
Infection and apoptosis in response to CHIKV E1:226 V infection. Summary of percentage infection (A) and apoptosis induction (B) in cell lines infected with CHIKV ECSA E1: 226 V (226 V) or mock infected (Mock).

A detailed profile of susceptibility to infection and the induction of apoptosis were determined for the ECSA E1: 226 V isolate for each of the cell lines. Cells were either mock infected or infected with CHIKV ECSA E1: 226 V at m.o.i. 1 and cells were subsequently analyzed on days 1, 2, 3 and 5 p.i. by flow cytometry after staining with either an anti-alphavirus monoclonal antibody followed by an appropriate FITC-labeled secondary antibody or with FITC-conjugated Annexin V and propidium iodide. Each experiment was undertaken independently in triplicate. Results for infection and apoptosis for all cell lines are summarized in Figure 3 and Table 1.

**Table 1:** Summary of susceptibility to infection and induction of apoptosis for three CHIKV isolates.

Cell line	Cell type	CHIKV ECSA (E1:226 V)		CHIKV ECSA (E1:A226)		CHIKV ECSA (ROSS)	
		Infection	Apoptosis	Infection	Apoptosis	Infection	Apoptosis
**CHME-5**	Human microglial	▴▴▴▴	▴▴▴▴	▴▴▴▴	▴▴▴▴	▴▴▴▴	▴▴▴▴
**HEK293T/17**	Human embryonic kidney	▴▴▴▴	▴▴▴▴	▴▴▴▴	▴▴▴▴	▴▴▴▴	▴▴▴▴
**Vero**	Monkey kidney	▴▴▴▴	▴▴▴	▴▴▴▴	▴▴▴	▴▴▴▴	▴▴▴▴
**HepG2**	Human hepatocellular	▴▴▴▴	▴▴▴▴	▴▴▴▴	▴▴▴▴	▴▴▴▴	▴▴▴▴
**Hela**	Human cervical epithelial	▴▴	▴▴	▴▴▴▴	▴▴▴▴	▴▴▴▴	▴▴▴▴
**MRC-5**	Human lung fibroblast	▴▴▴▴	▴	**ND**	**ND**	**ND**	**ND**
**SW-982**	Human synovial sarcoma	⁃	⁃	⁃	▴	▴	▴
**U937**	Human histiocytic lymphoma	⁃	⁃	⁃	⁃	⁃	⁃
**CCL-125**	*Aedes aegypti*	▴	**ND**	⁃	**ND**	⁃	**ND**
**C6/36**	*Aedes albopictus*	▴▴▴▴	⁃	▴▴▴▴	⁃	⁃	⁃

0%≤⁃≤20%; 21%≤▴≤40%; 41%≤▴▴≤60%; 61%≤▴▴▴≤80%; 81%≤▴▴▴▴≤100%; **ND**: Not determined.

Results showed that three cell lines namely HepG2 (Figure S1A), Vero (Figure S4A) and MRC-5 (Figure S6A) showed both infection and apoptosis rates of over 80% by 5 d.p.i. To directly visualize infection, Vero cells were either mock infected or infected with CHIKV E1: 226 V, and on days 1 and 2 p.i. stained with a primary anti-alphavirus monoclonal antibody followed by an appropriate FITC-conjugated secondary antibody as well as with TO-PRO-3 iodide to visualize nuclei before examination under a confocal microscope. Results (Figure S4C to F) confirmed high levels of infection of Vero on day 1 post infection (Figure S4A and Table 1) and by day 2 p.i. characteristic cellular blebs which stained positive for CHIKV were observed (Figure S4F) as previously seen by others [51].

Two further cell lines, CHME-5 (Figure 1A) and HEK293T/17 (Figure S3A) showed infection rates of over 80% by 5 d.p.i. but levels of apoptosis of approximately 60%. Hela infection levels reached a maximum of only some 30% of cells by day 5 p.i. and levels of induced apoptosis only reached about 25% on day 5 p.i. (Figure S2A and Table 1). Both SW-982 (Figure S5A) and U937 (Figure S7A) cells showed no appreciable levels of either infection or apoptosis. In contrast, C6/36 cells showed high levels of infection (>80%) from 1 d.p.i. but this level decreased dramatically to approximately 20% by 5 d.p.i. and occurred without the significant induction of apoptosis (Figure 2A).

### Comparison of ECSA E1: 226 V, ECSA E1:A226 and Ross strain

**Figure 4:**
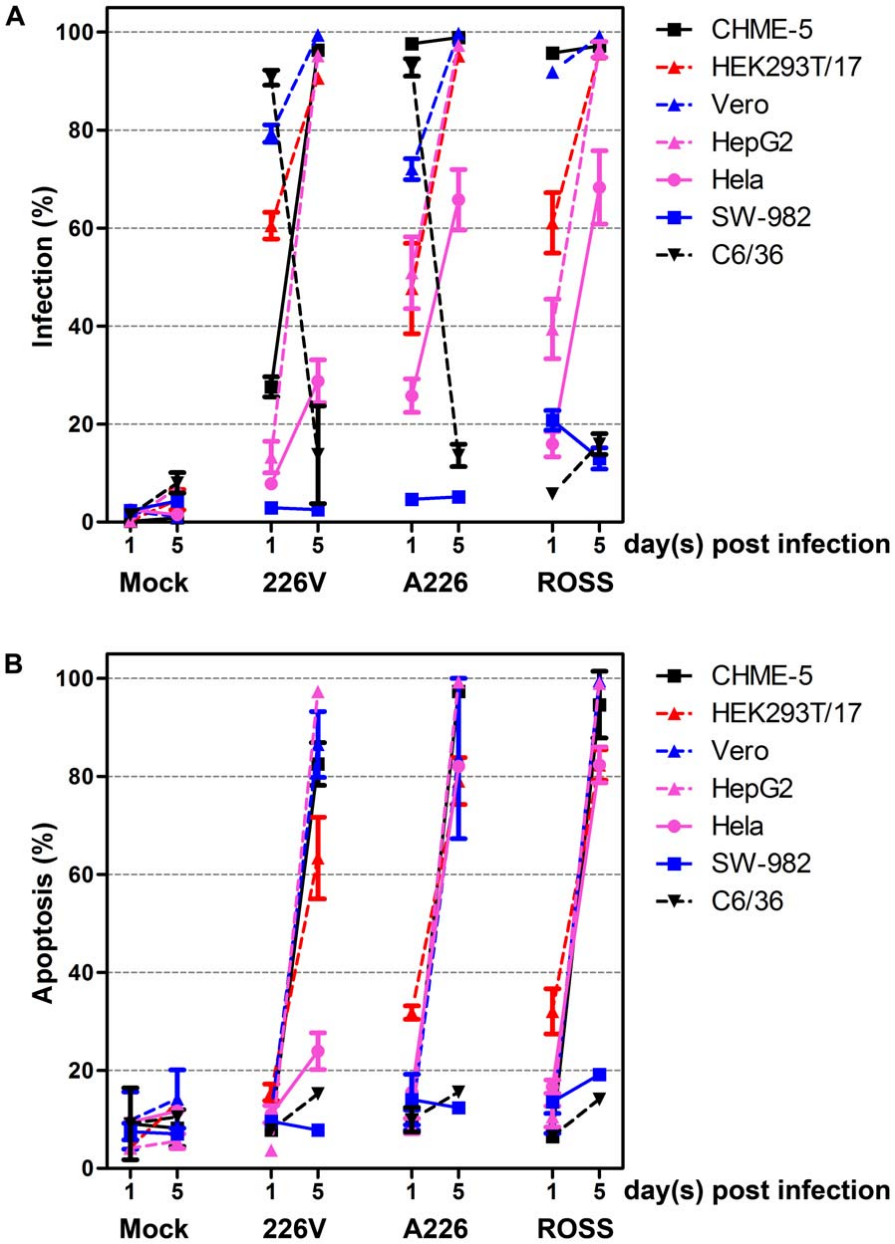
Comparative analysis of infection and apoptosis in response to CHIKV infection. Summary of percentage infection (A) and apoptosis induction (B) in cell lines infected with CHIKV ECSA E1:226 V (226 V), CHIKV ECSA E1: A226 (A226), Ross strain (ROSS) or mock infected (Mock).

CHME-5, HEK293T/17, HepG2, Hela, SW-982, U937, Vero, C6/36 and CCL-125 cells were either mock infected or infected separately with CHIKV ECSA E1: A226, ECSA E1: 226 V and Ross strain, and the cell susceptibility and induction of apoptosis examined on days 1 and 5 p.i. Because of technical difficulties with the MRC-5 cell line (the cells lost viability after the 5^th^ passage in our hands, and so only limited expansion was possible) this cell line was not included in the comparative analysis. Evaluation of susceptibility of U937 cells to all three CHIKV isolates showed no apparent infection of these cells with any isolate (Figure S7B and Table 1). In light of the negative result for infection, induction of apoptosis was not formally evaluated. Summarized results for all cell lines analyzed are shown in Figure 4.

CHME-5 (Figure 1B), HepG2 (Figure S1B), HEK293T/17 (Figure S3B) and Vero (Figure S4B) all showed comparable rates of infection and apoptosis in response to infection with all three isolates, although CHME-5 (Figure 1B) showed a much lower rate of infection on 1 d.p.i. with E1: 226 V as compared to the other two isolates (see also Figure 4 and Table 1). To directly visualize infection, CHME-5 cells were either mock infected or infected separately with all three CHIKV isolates, and on day 2 p.i. stained with a primary anti-alphavirus monoclonal antibodies followed by an appropriate FITC-conjugated secondary antibody as well as with TO-PRO-3 iodide to visualize nuclei before examination under a confocal microscope. Results (Figure 1C to F) confirmed high levels of infection with all three CHIKV isolates.

The E1: 226 V isolate showed lower rates of both infection and apoptosis of Hela cells as compared to the E1: A226 and Ross strain isolates (Figure S2B), while for the cell line SW-982 highest levels of infection were seen with Ross strain (Figure S5B and Table 1) although this reached a maximum of only 20%. Similarly, only a slight (maximum 20%) induction of apoptosis was observed (Figure S5B and Table 1). The low levels of infection were confirmed by immunocytochemistry using an anti-CHIKV antibody. Results showed only scattered infected cells for all three isolates on day 5 p.i. (Figure S5C to F).

Independent analysis of infection of C6/36 cells with all three CHIKV isolates is shown in Figure 2B and Table 1. Again, high levels of infection were seen on day 1 p.i. for CHIKV E1: A226 and CHIKV E1: 226 V which declined less than 20% on day 5 p.i. Ross strain however showed minimal infection of C6/36 cells on day 1, and a slight increase in infection by day 5 p.i. (Figures 2B). In all cases the number of apoptotic cells was comparable with mock infected cells.

To confirm this result, cells were infected with all three CHIKV isolates separately and examined by immunocytochemistry on days 1, 3 and 5 p.i. in parallel with mock infected cells. Results (Figure S8) showed high levels of CHIKV positive cells for CHIKV E1: A226 and CHIKV E1: 226 V on day 1 p.i. and only scattered minimally positive single cells by day 5 p.i. Ross strain showed significantly fewer infected cells on day 1 p.i. but more infected cells on day 3 p.i. as compared to the other two CHIKV isolates, however by day 5 p.i. there were also few positive cells (Figure S8).

The *Aedes aegypti* cell line CCL-125 was examined for infectivity with all three CHIKV isolate. As shown in Figure S9, while there were some infected cells on day 1 p.i. (approximately 20%) for the CHIKV E1: 226 V isolate, this declined over the following days. Infection of CCL-125 cells with the CHIKV E1: A226 isolate showed approximately 10% infection on day 1 which also declined over the following days. Surprisingly, no detectable infection of CCL-125 cells was seen with the Ross strain (Figure S9 and Table 1).

## Discussion

CHIKV has been present in Africa, Indian and many parts of Southeast Asia for many years [32], [33]. In Southeast Asia for example since the late 1960 s, CHIKV generally caused only relatively limited, small scale outbreaks [33], [52] although there was immunological evidence for exposure of local populations to the virus [53], [54]. However, the sudden and unpredicted outbreak of CHIKV that swept out of Africa and caused large epidemics in islands in the Indian Ocean and in the countries around the rim of the Indian Ocean served to refocus attention on this virus [34], [36], [55].

Epidemiological evidence showed that the massive outbreak has its roots in an outbreak of chikungunya fever in Kenya in 2004, and that outbreaks in India and Southeast Asia were driven by emergence of the ECSA lineage of CHIKV into areas previously affected by the Asian lineage of CHIKV [5], [32], [38]. While the epidemic of CHIKV infections was propelled by the ECSA lineage, analysis showed that the driving force behind the emergence of the virus was its adaptation to *Aedes albopictus* mosquitoes which occurred as a result of the emergence of ECSA viruses carrying the A226V substitution in the E1 structural protein of CHIKV [40], [42]. The size of the epidemic in La Reunion - which affected 40% of the population [34] , the autochthonous outbreak of chikungunya fever in Italy in 2007 [45], [46] and subsequently in France in 2010 [56] coupled with the increasing spread of *Aedes albopictus* mosquitoes [57] all served to fuel fear that populations previously at little or no risk of CHIKV infection could be at risk [58].

In our analysis of eight different cell lines representing a range of potential target cells, we found only minimal differences in the behavior of the three different CHIKV isolates, which were two recent isolates (CHIKV E1: A226, CHIKV E1: 226 V) and Ross strain representing the earliest genotype of the ECSA lineage. In both the spectrum of cells able to be infected, as well as the degree of infection and the induction of apoptosis there were only marginal differences between these isolates.

The two cell lines where some differences were observed in the interaction of the three isolates were SW-982 synovial cells and Hela cervical epithelial cells. The SW-982 cell line presented an anomalous pattern of virus titer as compared to the other cell lines. In cells that were susceptible to infection there was an immediate drop in virus titer followed by *de novo* production at a later time point as evidenced by an increasing titer, while the non-susceptible U937 cell line showed no drop in titer during the initial infection. SW-982 cells showed the drop in titer characteristic of susceptible cells, but infectivity remained extremely low, suggesting that the main block virus production in this cell line is not at the virus binding/internalization step. Additionally, Ross showed the highest level of infection of the three isolates in this cell line. However, while arthralgia is one of the main presenting symptoms of chikungunya infection [35], [59], the direct involvement of synovial cells in the disease pathology remains to be established.

Hela cells have previously been shown to be susceptible to CHIKV and the level of apoptosis seen with the ECSA CHIKV E1: 226 V isolate (about 30%) was consistent with previous reports which utilized a similar isolate [51]. Interestingly however, both Ross strain (which is also alanine at E1: 226) and the recent ECSA CHIKV E1: A226 isolate showed much higher levels of infection and apoptosis than the ECSA E1: 226 V.

The most significant difference observed between these isolates was in their interaction with insect cells. While both the ECSA E1: A226 and ECSA E1: 226 V isolates showed very high levels of infectivity on day 1 p.i. the Ross strain isolate showed surprisingly low levels (<10%) on the same day. This suggests that much of the increased adaptation of the recent ECSA E1: 226 V lineage is also shared by the E1: A226 isolate, at least in comparison to the Ross strain, which suggests that the increased adaptability of the newer isolates is the ultimate result of a longer series of changes in the ECSA genome. In support of this, some studies have suggested that the effect of the E1: A226V substitution is modulated by other changes in the CHIKV genome, in particularly in the structural E2 protein [60]. In the *Aedes aegypti* CCL-125 cell line, none of the isolates showed a high level of infection, although the ECSA E1: 226 V isolate showed the highest level of infectivity, suggesting that this isolate may also be more suited to replication in both *Aedes albopictus* and *Aedes aegypti* as has been suggested by others [40], [42].

Remarkably however, in both cell lines an almost complete clearance of the virus was observed by day 5 for all three isolates. While RNA interference (RNAi) in which long double stranded RNA molecules are cleaved into small RNA effector molecules by Dicer and which subsequently silence complimentary RNA sequences [61] is the primary insect cell defense pathway against invading viruses [62], this pathway is not active in C6/36 cells [63] and as such cannot explain the mechanism by which CHIKV is cleared from these cells. However activation of the RNAi pathway may explain the extremely limited nature of infection seen in the *Aedes aegypti* CCL-125 cells.

Overall therefore our results show little evolution of CHIKV infectivity or cytopathogenicity in mammalian cells. The biggest difference between the prototype CHIKV Ross strain and contemporary isolates was in the infectivity in insect cells, particularly in *Aedes albopictus* cells, which supports the proposal that the current spread of the ECSA is largely driven by increased fitness of the ECSA lineage in insect vectors [42], [44].

## Materials and Methods

### Ethics statement

The virus isolation described in the relevant section (virus isolation and propagation) was part of a study approved by the Mahidol University Institutional Review Board (COA. NO. MU-IRB 2010/251.3018) and by the Ethics Review Board of Pang Nga Hospital, Thailand. Written informed consent was obtained.

### Cells and culture conditions

The embryonic fetal human microglial cell line CHME-5 [64] was kindly provided by Professor Pierre Talbot, Laboratory of Neuroimmunovirology, INRS-Institute, Armand-Frappier, Canada. The human lung fibroblast cell line MRC-5 [65] was kindly provided by Assistant Professor Arunee Thitithanyanont, Microbiology, Faculty of Science, Mahidol University. The human embryonic kidney cell line HEK293T/17 (ATCC Cat No. CRL-11268) [66], the human synovial sarcoma cell line SW-982 (ATCC Cat No. HTB-93), the human histiocytic lymphoma cell line U937 (ATCC Cat No. CRL-1593.2) [67] and the *Aedes aegypti* cell line CCL-125 [50] were obtained from ATCC (American Type Culture Collection, Manassas, VA). CHME-5, HEK293T/17, HepG2, MRC-5, Hela (ATCC Cat No. CCL-2) and SW-982 cells were cultured at 37°C, 5% CO_2_ in Dulbecco's modified Eagle's medium (DMEM; Gibco, Invitrogen, Carlsbad, CA) supplemented with 10% heat-inactivated fetal bovine serum (FBS; Gibco, Invitrogen) and 100 units of penicillin and 100 µg streptomycin/ml while Vero (ATCC Cat No. CCL-81) and BHK-21 (ATCC Cat. No. CCL-10) were supplemented with 5% FBS. U937 [67] cells were cultured at 37°C, 5% CO_2_ in RPMI-1640 (Gibco, Invitrogen) supplemented with 10% FBS and 100 units of penicillin and 100 µg streptomycin/ml. C6/36 cells [50] were cultured at 28°C in MEM (Gibco, Invitrogen) 10% FBS and 100 units of penicillin and 100 µg streptomycin/ml whereas CCL-125 cells [50] were cultured in Leibovitz L-*15* medium (Gibco, Invitrogen) 20% FBS and 100 units of penicillin and 100 µg streptomycin/ml.

### Virus isolation and propagation

The CHIKV East, Central and South African (ECSA) genotype E1: 226V was isolated from a patient in Phang Nga Province, Thailand in 2009 and was passaged once through C6/36 cells followed by 3 passages through passage through Vero cells. The virus isolation was part of a study was approved by the Mahidol University Institutional Review Board (COA. NO. MU-IRB 2010/251.3018) and by the Ethics Review Board of Pang Nga Hospital, Thailand. Written informed consent was obtained. The CHIKV ECSA genotype E1: A226 was a kind gift from Drs Duane Gubler and Eng-Eong Ooi of the Emerging Infectious Disease Program, Duke-NUS Medical School, Singapore. It was passaged through BHK-21 cells 3 times followed by passage twice through Vero cells. The CHIKV ECSA Ross genotype (Ross strain, E1: A226) was passaged 5 times through Vero cells.

### Virus infection

CHME-5, HEK293T/17, HepG2, MRC-5, Hela, SW-982, U937, Vero, C6/36 and CCL-125 cells were plated into 6-well plates and grown until cells reach approximately 80% confluency after which cells were infected with CHIKV at a multiplicity of infection (m.o.i.) of 1, for 2 h. After infection, fresh medium containing serum was added to the cells and the cells were incubated under standard conditions. Culture medium was supplemented everyday to avoid nutrient deprivation. Infected cells and culture medium were collected daily. At each time point, infected cells were quantified by flow cytometry in parallel with observation of the induction of apoptosis by FITC-conjugated Annexin V and propidium iodide staining. The experiment was undertaken independently in triplicate.

### Flow cytometry

To analyze apoptosis, cells were collected and washed with ice-cold PBS and were resuspended in binding buffer (BD, Franklin Lakes, NJ), followed by double staining with the addition of 50 µg/ml FITC-conjugated Annexin V and 20 µg/ml propidium iodide. After 15 min, the cells were analyzed by flow cytometry (BD, FACSCalibur).

To quantify the infected cells, total cells were harvested and blocked with 10% normal goat serum for 30 min on ice. Cells were washed with 1% BSA follow by fixation in 4% paraformaldehyde at room temperature for 20 min. Subsequently cells were permeabilized with 0.2% Triton X-100 in 1% BSA for 10 min at room temperature. Cells were then incubated with a mouse anti-alphavirus monoclonal antibody (Santa Cruz Biotechnology Inc., Santa Cruz, CA) at a dilution of 1∶200 at 4°C for overnight. After three washes with 1% BSA, cells were incubated with a FITC conjugated goat anti-mouse IgG polyclonal antibody (KPL, Gaithersburg, MD) at dilution of 1∶20 at room temperature for 1 h. Cells were washed three times with 1% BSA and resuspended with PBS and analyzed by flow cytometry (BD, FACSCalibur) using the CELLQuest™ software (BD Biosciences).

### Indirect immunofluorescence microscopy

Cells were grown on cover slips and further infected with each CHIKV at m.o.i. of 1 as previously described. At each time point, cells were fixed with ice-cold methanol for 20 min. After washed with PBS, cells were then permeabilized with 0.2% Triton X-100 in PBS for 10 min at room temperature before incubating with 10% normal goat serum in PBS for 1 h at room temperature. Subsequently, cells were incubated with a mouse anti-alphavirus monoclonal antibody (Santa Cruz Biotechnology Inc.) at a dilution of 1∶200 at 4°C for overnight. After washing, cells were incubated with a 1∶20 dilution of a FITC-conjugated goat anti-mouse IgG polyclonal antibody (KPL, USA) and 4 µM of TO-PRO-3 iodide (Gibco, Invitrogen) for 1 h at room temperature before the observation under an Olympus FluoView 1000 confocal microscope.

### Standard plaque assay

Vero cell were plated into 6-well plate until the confluency reached up to approximately 100 percent. Cells were inoculated with ten-fold dilution of culture supernatant containing infectious viruses and incubated for 2 h at 37°C with constant agitation. Subsequently 0.8% Seakem Le agarose (Cambrex, USA) mixed with nutrient overlay (Earle's Balanced Salts supplemented with 0.033% (w/v) yeast extract, 0.165% lactalbumin hydrolysate, 3% FBS) was added to each well. The plates were incubated for a further 3 days at 37°C before 0.8% Seakem Le agarose mixed with second nutrient overlay (Earle's Balanced Salts supplemented with 0.063% (w/v) yeast extract, 0.315% lactalbumin hydrolysate and 0.035% (w/v) Neutral red) was added to each well. Plaques were count after incubating the plates for a further 15–24 h at 37°C. Each experiment was done independently in triplicate, with duplicate assay of titer.

## Supporting Information

Figure S1 **CHIKV infection of HepG2 human hepatocellular carcinoma cells.** A. HepG2 cells were mock infected (M) or infected with CHIKV ECSA E1: 226 V (EV) CHIKV at m.o.i. of 1. Infected cells and culture medium were collected daily and the medium assayed for levels of infectious CHIKV at the times indicated by standard plaque assay on Vero cells, while cells were assayed for infectivity and induction of apoptosis by flow cytometry after staining with an anti-alphavirus monoclonal antibody and FITC-conjugated Annexin V/propidium iodide respectively. B. HepG2 cells were mock infected (M) or infected with ECSA CHIKV E1: A226 (EA), ECSA CHIKV E1: 226 V (EV) or Ross strain (RO) at m.o.i. of 1 and assayed for infectivity and induction of apoptosis by flow cytometry on the days indicated. All experiments were undertaken independently in triplicate with duplicate analysis of virus titers. Error bars show S.D.
(TIF)

Figure S2 **CHIKV infection of Hela human cervical carcinoma cells.** A. Hela cells were mock infected (M) or infected with CHIKV ECSA E1: 226 V (EV) CHIKV at m.o.i. of 1. Infected cells and culture medium were collected daily and the medium assayed for levels of infectious CHIKV at the times indicated by standard plaque assay on Vero cells, while cells were assayed for infectivity and induction of apoptosis by flow cytometry after staining with an anti-alphavirus monoclonal antibody and FITC-conjugated Annexin V/propidium iodide respectively. B. Hela cells were mock infected (M) or infected with ECSA CHIKV E1: A226 (EA), ECSA CHIKV E1: 226 V (EV) or Ross strain (RO) at m.o.i. of 1 and assayed for infectivity and induction of apoptosis by flow cytometry on the days indicated. All experiments were undertaken independently in triplicate with duplicate analysis of virus titers. Error bars show S.D.
(TIF)

Figure S3 **CHIKV infection of HEK293T/17 human fetal kidney cells.** A. HEK293T/17 cells were mock infected (M) or infected with CHIKV ECSA E1: 226 V (EV) CHIKV at m.o.i. of 1. Infected cells and culture medium were collected daily and the medium assayed for levels of infectious CHIKV at the times indicated by standard plaque assay on Vero cells, while cells were assayed for infectivity and induction of apoptosis by flow cytometry after staining with an anti-alphavirus monoclonal antibody and FITC-conjugated Annexin V/propidium iodide respectively. B. HEK293T/17 cells were mock infected (M) or infected with ECSA CHIKV E1: A226 (EA), ECSA CHIKV E1: 226 V (EV) or Ross strain (RO) at m.o.i. of 1 and assayed for infectivity and induction of apoptosis by flow cytometry on the days indicated. All experiments were undertaken independently in triplicate with duplicate analysis of virus titers. Error bars show S.D.
(TIF)

Figure S4 **CHIKV infection of Vero monkey kidney cells.** A. Vero cells were mock infected (M) or infected with CHIKV ECSA E1: 226 V (EV) at m.o.i. of 1. Infected cells and culture medium were collected daily and the medium assayed for levels of infectious CHIKV at the times indicated by standard plaque assay on Vero cells, while cells were assayed for infectivity and induction of apoptosis by flow cytometry after staining with an anti-alphavirus monoclonal antibody and FITC-conjugated Annexin V/propidium iodide respectively. B. Vero cells were mock infected (M) or infected with ECSA CHIKV E1: A226 (EA), ECSA CHIKV E1: 226 V (EV) or Ross strain (RO) at m.o.i. of 1 and assayed for infectivity and induction of apoptosis by flow cytometry on the days indicated. All experiments were undertaken independently in triplicate with duplicate analysis of virus titers. Error bars show S.D. C to F. Vero cells were mock infected (C, E) or infected with ECSA CHIKV E1: 226 V (D, F), and on days 1 (C, D) and 2 (E, F) p.i. stained with a mouse anti-alphavirus monoclonal antibody followed by a FITC conjugated goat anti-mouse IgG polyclonal antibody (green). Nuclei of cells were stained with TO-PRO-3 iodide (red). Non-contrast adjusted merged images are shown.
(TIF)

Figure S5 **CHIKV infection of SW-982 human synovial sarcoma cells.** A. SW-982 cells were mock infected (M) or infected with CHIKV ECSA E1: 226 V (EV) at m.o.i. of 1. Infected cells and culture medium were collected daily and the medium assayed for levels of infectious CHIKV at the times indicated by standard plaque assay on Vero cells, while cells were assayed for infectivity and induction of apoptosis by flow cytometry after staining with an anti-alphavirus monoclonal antibody and FITC-conjugated Annexin V/propidium iodide respectively. B. SW-982 cells were mock infected (M) or infected with ECSA CHIKV E1: A226 (EA), ECSA CHIKV E1: 226 V (EV) or Ross strain (RO) at m.o.i. of 1 and assayed for infectivity and induction of apoptosis by flow cytometry on the days indicated. All experiments were undertaken independently in triplicate with duplicate analysis of virus titers. Error bars show S.D. C to F. SW982 cells were mock infected (C) or infected with ECSA CHIKV E1: 226 V (D), ECSA CHIKV E1: A226 (E) or Ross strain (F) and on day 5 p.i. stained with a mouse anti-alphavirus monoclonal antibody followed by a FITC conjugated goat anti-mouse IgG polyclonal antibody (green). Nuclei of cells were stained with TO-PRO-3 iodide (red). Non-contrast adjusted merged images are shown.
(TIF)

Figure S6 **CHIKV infection of MRC-5 human lung fibroblast cells.** MRC-5 cells were mock infected (M) or infected with CHIKV ECSA E1: 226 V (EV) at m.o.i. of 1. Infected cells and culture medium were collected daily and the medium assayed for levels of infectious CHIKV at the times indicated by standard plaque assay on Vero cells, while cells were assayed for infectivity and induction of apoptosis by flow cytometry after staining with an anti-alphavirus monoclonal antibody and FITC-conjugated Annexin V/propidium iodide respectively. All experiments were undertaken independently in triplicate with duplicate analysis of virus titers. Error bars show S.D.
(TIF)

Figure S7 **CHIKV infection of U937 human monocytic cells.** U937 cells were mock infected (M) or infected with CHIKV ECSA E1: 226 V (EV) at m.o.i. of 1. Infected cells and culture medium were collected daily and the medium assayed for levels of infectious CHIKV at the times indicated by standard plaque assay on Vero cells, while cells were assayed for infectivity and induction of apoptosis by flow cytometry after staining with an anti-alphavirus monoclonal antibody and FITC-conjugated Annexin V/propidium iodide respectively. B. U937 cells were mock infected (M) or infected with ECSA CHIKV E1: A226 (EA), ECSA CHIKV E1: 226 V (EV) or Ross strain (RO) at m.o.i. of 1 and assayed for infectivity and induction of apoptosis by flow cytometry on the days indicated. All experiments were undertaken independently in triplicate with duplicate analysis of virus titers. Error bars show S.D.
(TIF)

Figure S8 **Analysis of CHIKV infected C6/36 cells.** C6/36 cells were mock infected (A to C) or infected with CHIKV E1: 226 V (D to F), CHIKV E1: A226 (G to I) or Ross strain (J to L) at m.o.i. of 1 and subsequently stained with a mouse anti-alphavirus monoclonal antibody followed by a FITC conjugated goat anti-mouse IgG polyclonal antibody (green). Nuclei of cells were stained with TO-PRO-3 iodide (red) on days 1 (A, D, G and J), 3 (B, E, H and K) and 5 (C, F, I and L) p.i. Non-contrast adjusted merged images are shown.
(TIF)

Figure S9 **CHIKV infection of CCL-125** ***Aedes aegypti*** **cells.** A. CCL-125 cells were mock infected (M) or infected with CHIKV ECSA E1: 226 V (EV) at m.o.i. of 1. Infected cells and culture medium were collected daily and assayed for infectivity by flow cytometry after staining with an anti-alphavirus monoclonal antibody.
(TIF)
