# Supplementary figures and images for: Chikungunya Virus Infection of Cell Lines: Analysis of the East, Central and South African Lineage
Source: PLoS One. 2012 Jan 27;7(1):e31102. doi: 10.1371/journal.pone.0031102 (PMC3267766; doi:10.1371/journal.pone.0031102)

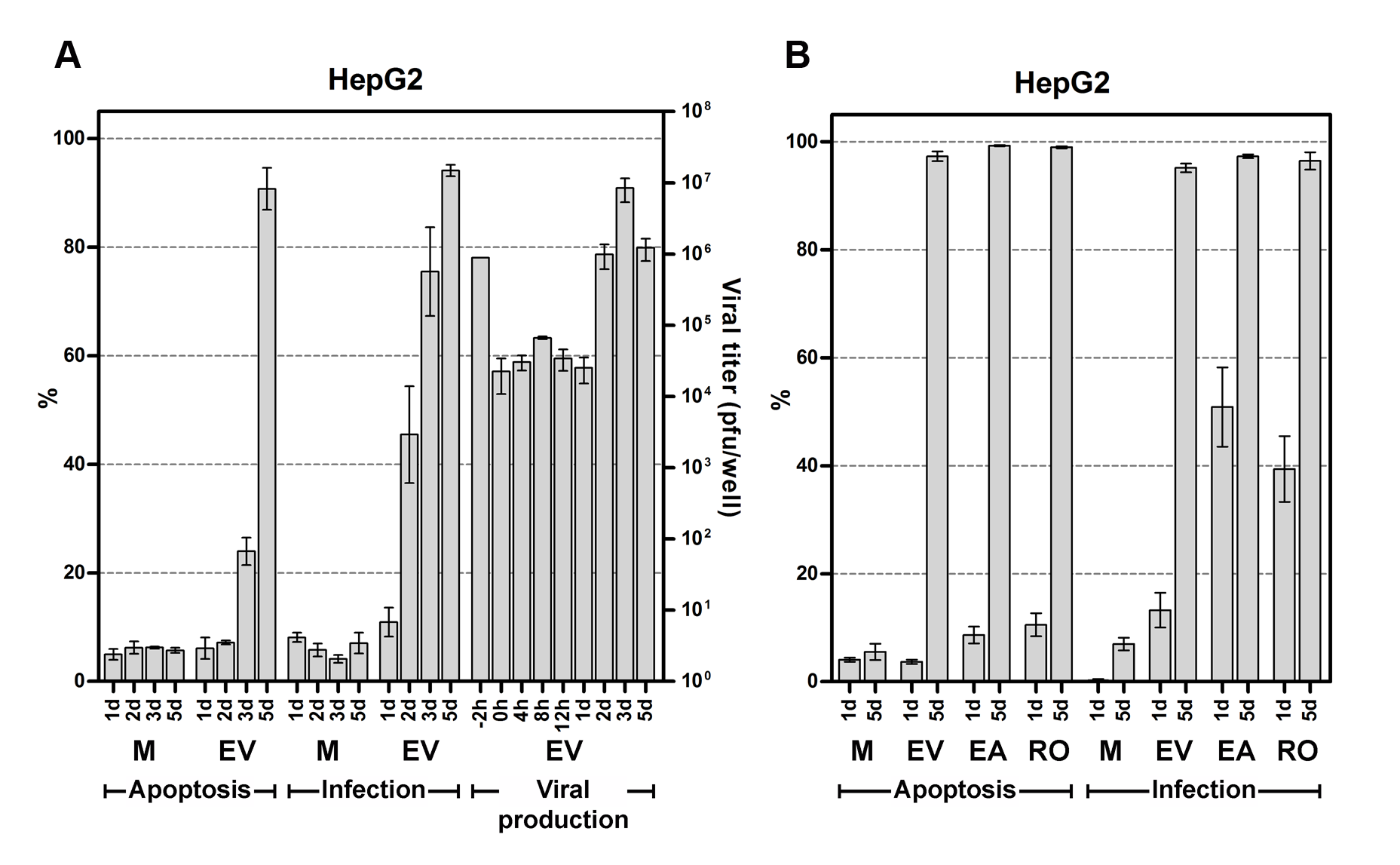

Supplement: Figure S1 — CHIKV infection of HepG2 human hepatocellular carcinoma cells. A. HepG2 cells were mock infected (M) or infected with CHIKV ECSA E1: 226 V (EV) CHIKV at m.o.i. of 1. Infected cells and culture medium were collected daily and the medium assayed for levels of infectious CHIKV at the times indicated by standard plaque assay on Vero cells, while cells were assayed for infectivity and induction of apoptosis by flow cytometry after staining with an anti-alphavirus monoclonal antibody and FITC-conjugated Annexin V/propidium iodide respectively. B. HepG2 cells were mock infected (M) or infected with ECSA CHIKV E1: A226 (EA), ECSA CHIKV E1: 226 V (EV) or Ross strain (RO) at m.o.i. of 1 and assayed for infectivity and induction of apoptosis by flow cytometry on the days indicated. All experiments were undertaken independently in triplicate with duplicate analysis of virus titers. Error bars show S.D. (TIF) [file pone.0031102.s001.tif]

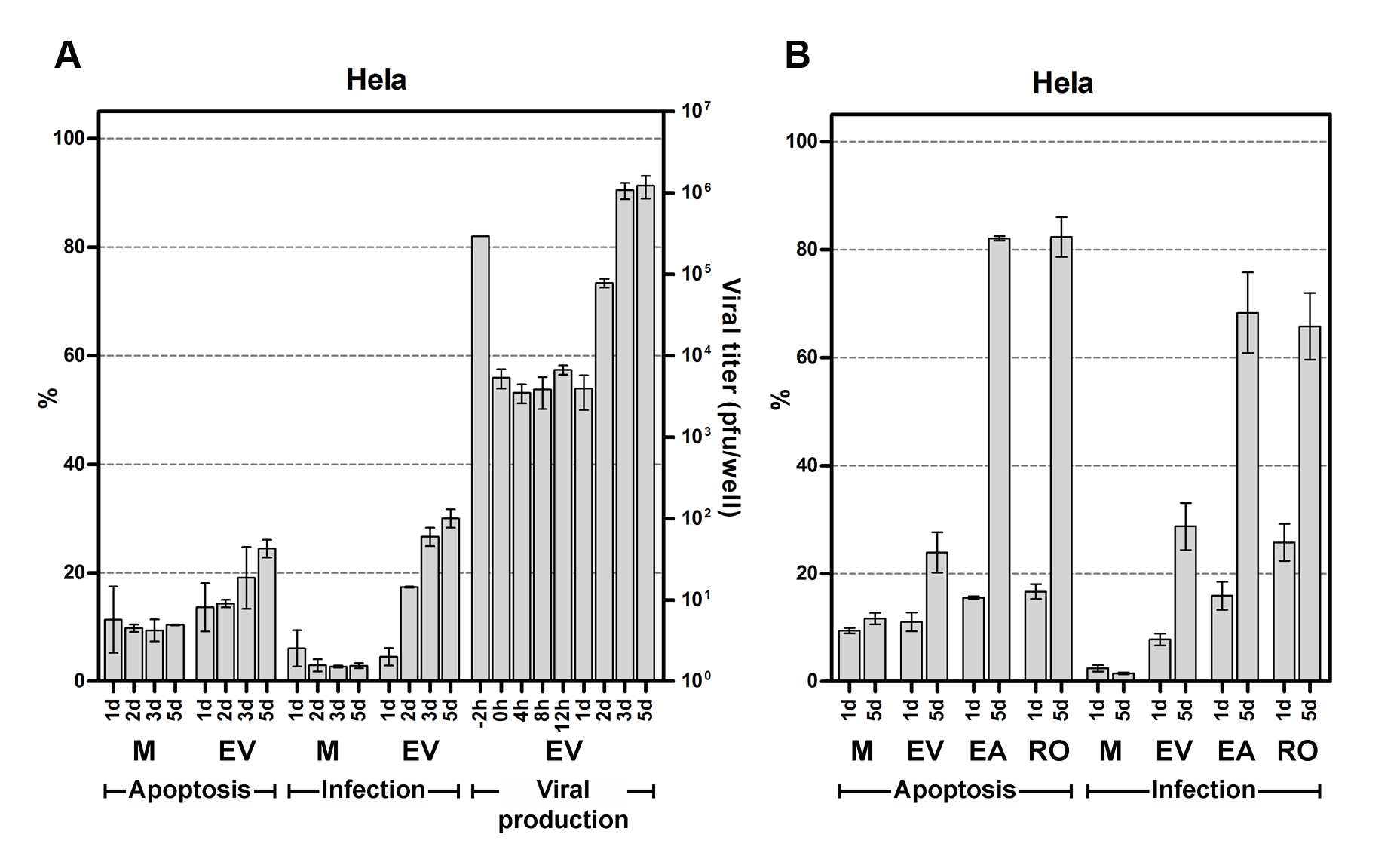

Supplement: Figure S2 — CHIKV infection of Hela human cervical carcinoma cells. A. Hela cells were mock infected (M) or infected with CHIKV ECSA E1: 226 V (EV) CHIKV at m.o.i. of 1. Infected cells and culture medium were collected daily and the medium assayed for levels of infectious CHIKV at the times indicated by standard plaque assay on Vero cells, while cells were assayed for infectivity and induction of apoptosis by flow cytometry after staining with an anti-alphavirus monoclonal antibody and FITC-conjugated Annexin V/propidium iodide respectively. B. Hela cells were mock infected (M) or infected with ECSA CHIKV E1: A226 (EA), ECSA CHIKV E1: 226 V (EV) or Ross strain (RO) at m.o.i. of 1 and assayed for infectivity and induction of apoptosis by flow cytometry on the days indicated. All experiments were undertaken independently in triplicate with duplicate analysis of virus titers. Error bars show S.D. (TIF) [file pone.0031102.s002.tif]

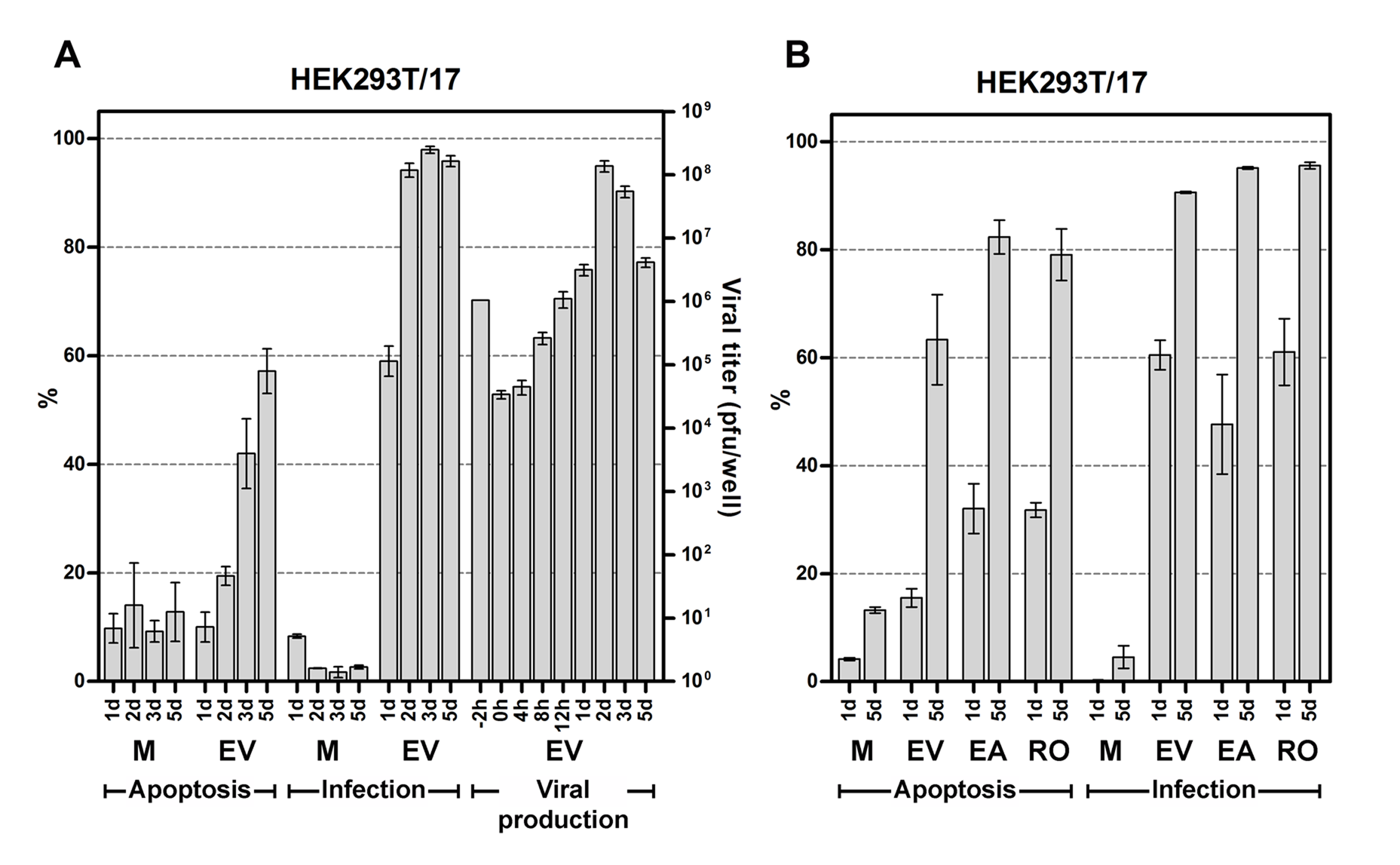

Supplement: Figure S3 — CHIKV infection of HEK293T/17 human fetal kidney cells. A. HEK293T/17 cells were mock infected (M) or infected with CHIKV ECSA E1: 226 V (EV) CHIKV at m.o.i. of 1. Infected cells and culture medium were collected daily and the medium assayed for levels of infectious CHIKV at the times indicated by standard plaque assay on Vero cells, while cells were assayed for infectivity and induction of apoptosis by flow cytometry after staining with an anti-alphavirus monoclonal antibody and FITC-conjugated Annexin V/propidium iodide respectively. B. HEK293T/17 cells were mock infected (M) or infected with ECSA CHIKV E1: A226 (EA), ECSA CHIKV E1: 226 V (EV) or Ross strain (RO) at m.o.i. of 1 and assayed for infectivity and induction of apoptosis by flow cytometry on the days indicated. All experiments were undertaken independently in triplicate with duplicate analysis of virus titers. Error bars show S.D. (TIF) [file pone.0031102.s003.tif]

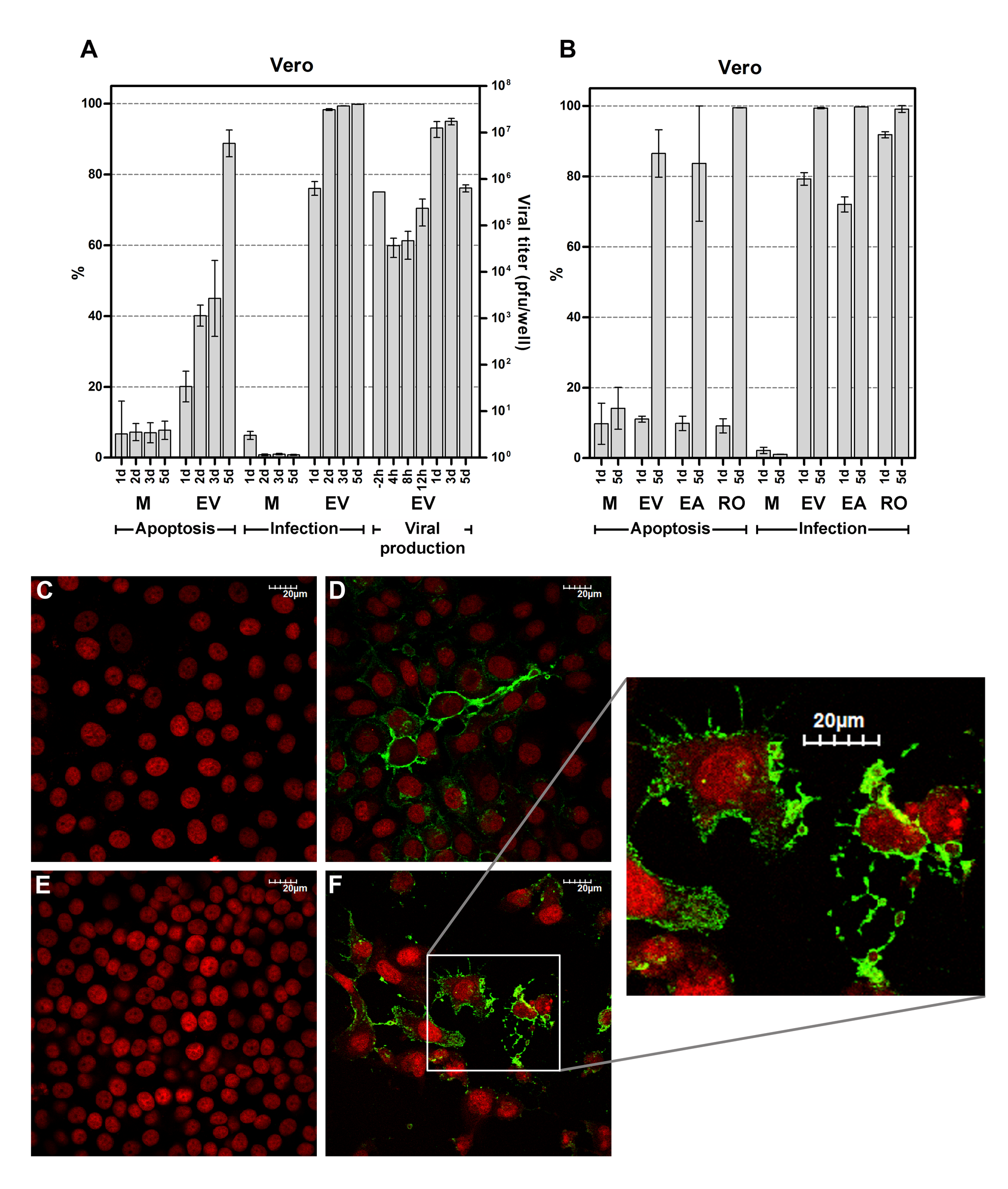

Supplement: Figure S4 — CHIKV infection of Vero monkey kidney cells. A. Vero cells were mock infected (M) or infected with CHIKV ECSA E1: 226 V (EV) at m.o.i. of 1. Infected cells and culture medium were collected daily and the medium assayed for levels of infectious CHIKV at the times indicated by standard plaque assay on Vero cells, while cells were assayed for infectivity and induction of apoptosis by flow cytometry after staining with an anti-alphavirus monoclonal antibody and FITC-conjugated Annexin V/propidium iodide respectively. B. Vero cells were mock infected (M) or infected with ECSA CHIKV E1: A226 (EA), ECSA CHIKV E1: 226 V (EV) or Ross strain (RO) at m.o.i. of 1 and assayed for infectivity and induction of apoptosis by flow cytometry on the days indicated. All experiments were undertaken independently in triplicate with duplicate analysis of virus titers. Error bars show S.D. C to F. Vero cells were mock infected (C, E) or infected with ECSA CHIKV E1: 226 V (D, F), and on days 1 (C, D) and 2 (E, F) p.i. stained with a mouse anti-alphavirus monoclonal antibody followed by a FITC conjugated goat anti-mouse IgG polyclonal antibody (green). Nuclei of cells were stained with TO-PRO-3 iodide (red). Non-contrast adjusted merged images are shown. (TIF) [file pone.0031102.s004.tif]

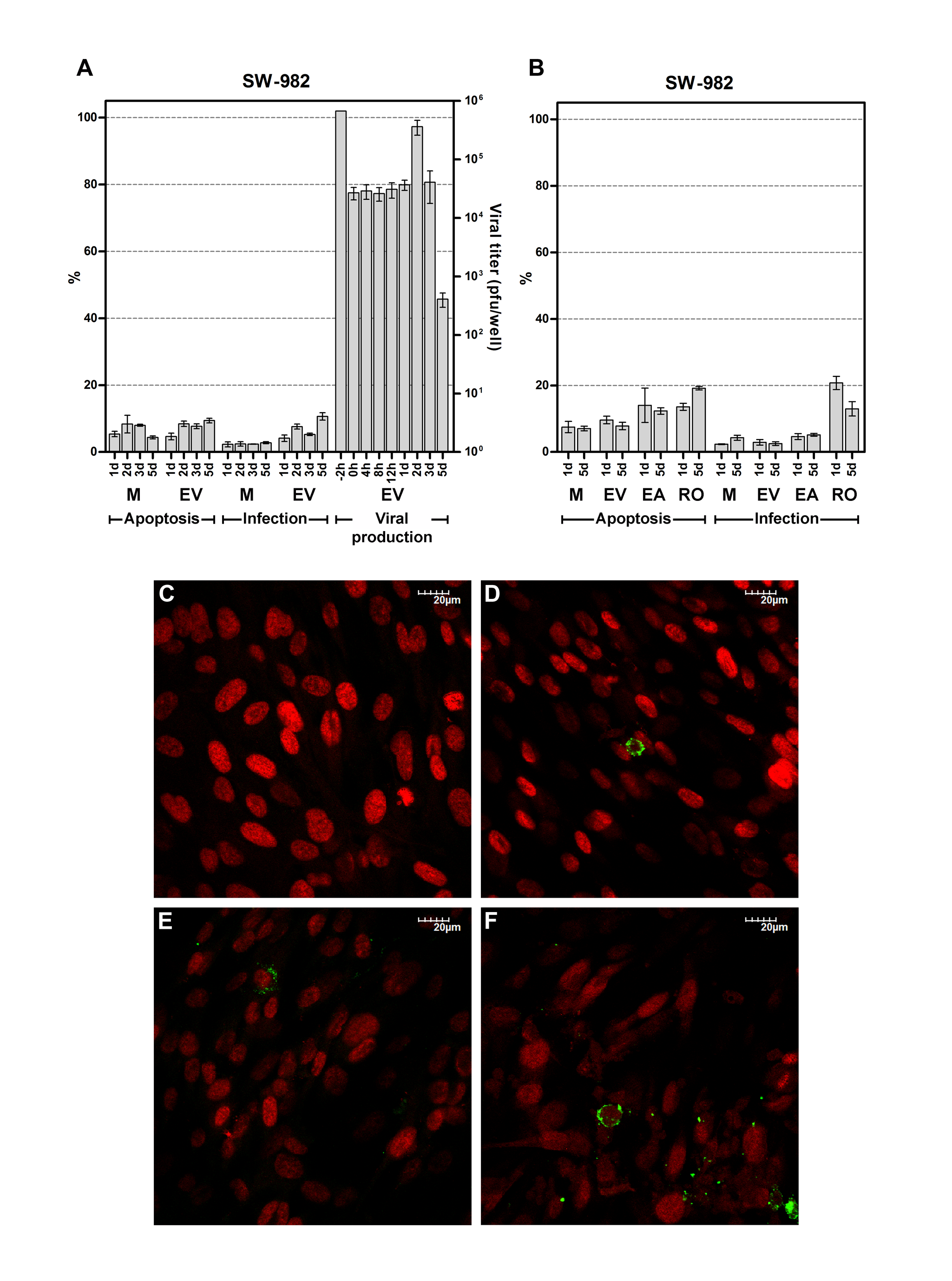

Supplement: Figure S5 — CHIKV infection of SW-982 human synovial sarcoma cells. A. SW-982 cells were mock infected (M) or infected with CHIKV ECSA E1: 226 V (EV) at m.o.i. of 1. Infected cells and culture medium were collected daily and the medium assayed for levels of infectious CHIKV at the times indicated by standard plaque assay on Vero cells, while cells were assayed for infectivity and induction of apoptosis by flow cytometry after staining with an anti-alphavirus monoclonal antibody and FITC-conjugated Annexin V/propidium iodide respectively. B. SW-982 cells were mock infected (M) or infected with ECSA CHIKV E1: A226 (EA), ECSA CHIKV E1: 226 V (EV) or Ross strain (RO) at m.o.i. of 1 and assayed for infectivity and induction of apoptosis by flow cytometry on the days indicated. All experiments were undertaken independently in triplicate with duplicate analysis of virus titers. Error bars show S.D. C to F. SW982 cells were mock infected (C) or infected with ECSA CHIKV E1: 226 V (D), ECSA CHIKV E1: A226 (E) or Ross strain (F) and on day 5 p.i. stained with a mouse anti-alphavirus monoclonal antibody followed by a FITC conjugated goat anti-mouse IgG polyclonal antibody (green). Nuclei of cells were stained with TO-PRO-3 iodide (red). Non-contrast adjusted merged images are shown. (TIF) [file pone.0031102.s005.tif]

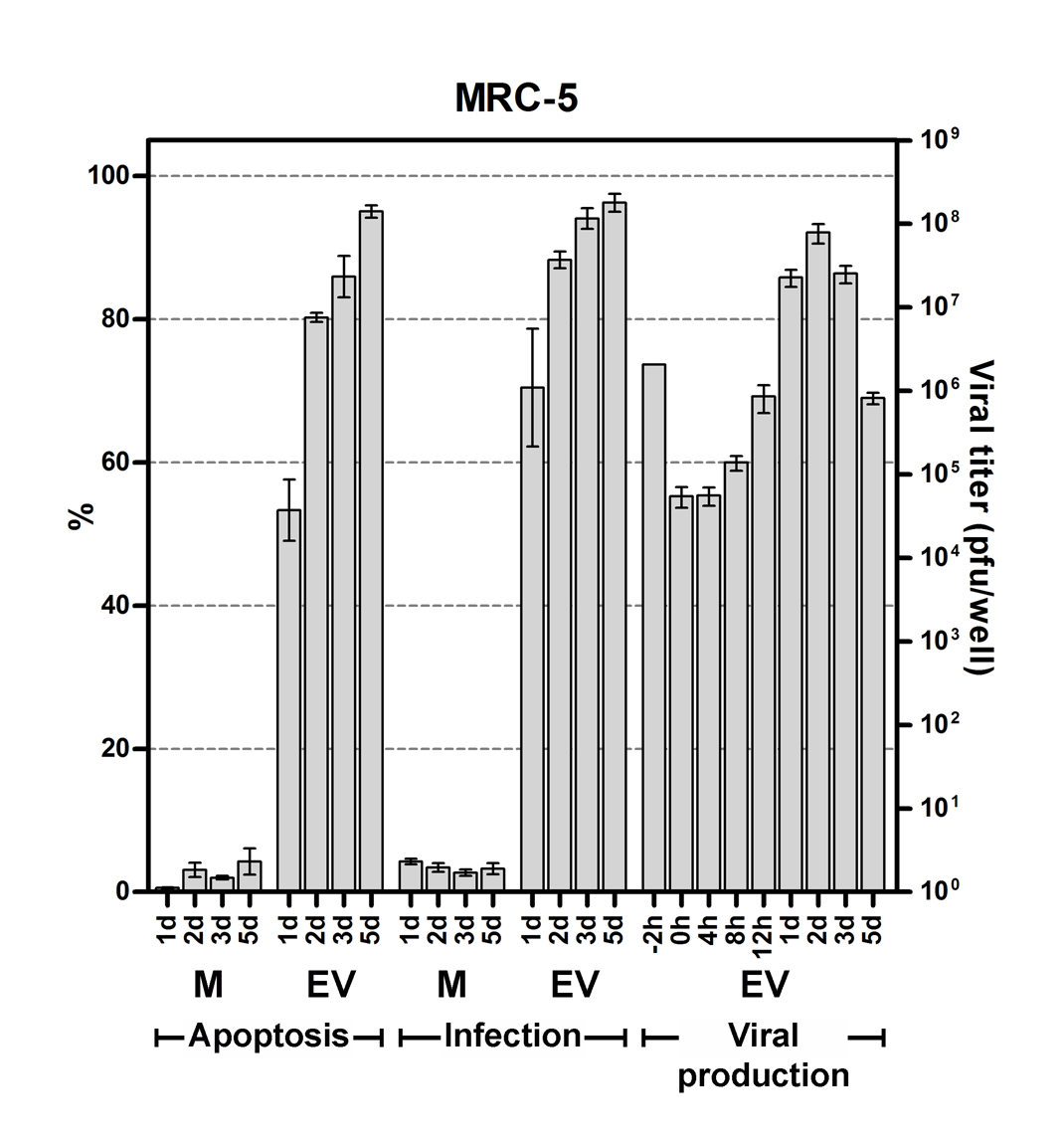

Supplement: Figure S6 — CHIKV infection of MRC-5 human lung fibroblast cells. MRC-5 cells were mock infected (M) or infected with CHIKV ECSA E1: 226 V (EV) at m.o.i. of 1. Infected cells and culture medium were collected daily and the medium assayed for levels of infectious CHIKV at the times indicated by standard plaque assay on Vero cells, while cells were assayed for infectivity and induction of apoptosis by flow cytometry after staining with an anti-alphavirus monoclonal antibody and FITC-conjugated Annexin V/propidium iodide respectively. All experiments were undertaken independently in triplicate with duplicate analysis of virus titers. Error bars show S.D. (TIF) [file pone.0031102.s006.tif]

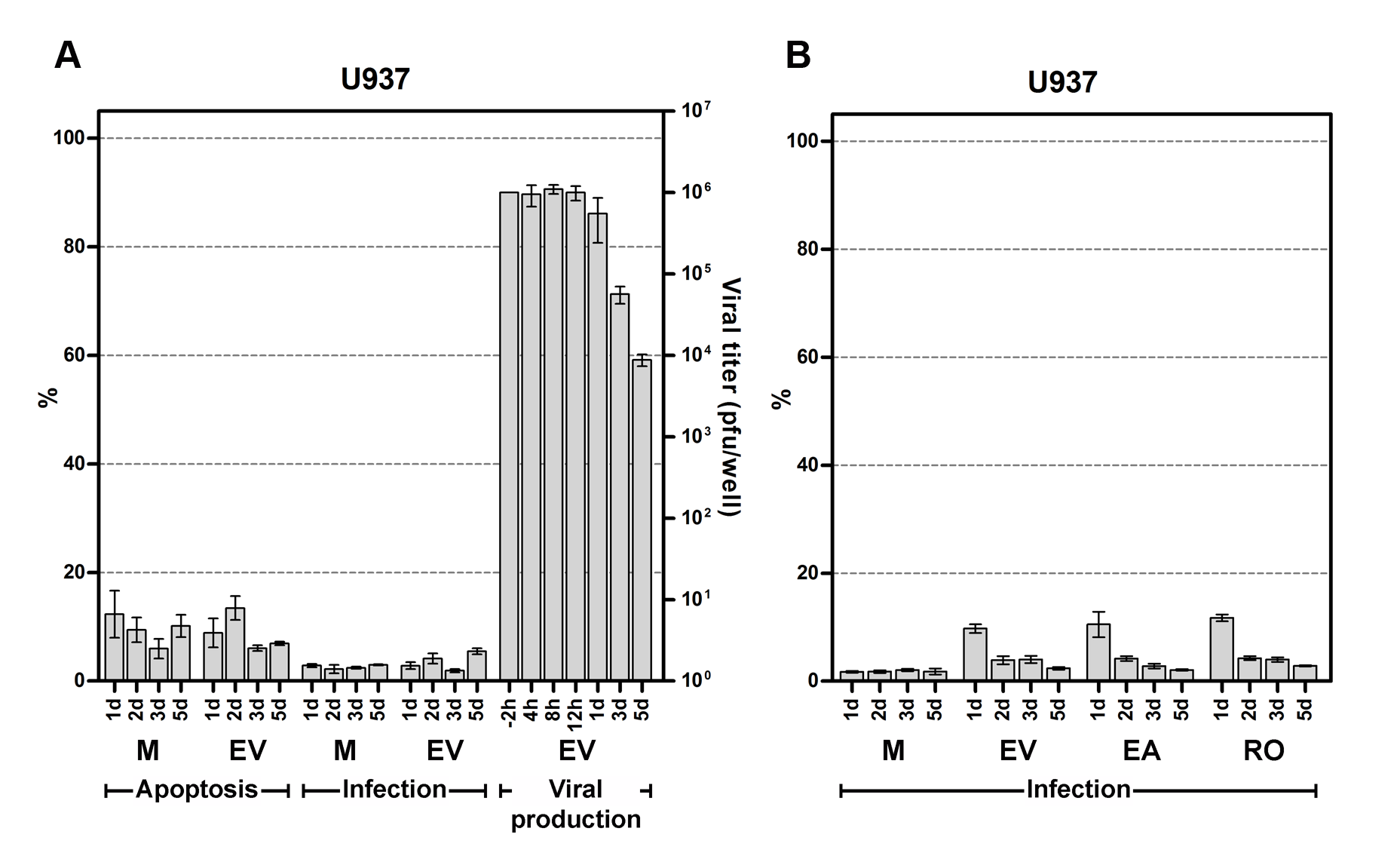

Supplement: Figure S7 — CHIKV infection of U937 human monocytic cells. U937 cells were mock infected (M) or infected with CHIKV ECSA E1: 226 V (EV) at m.o.i. of 1. Infected cells and culture medium were collected daily and the medium assayed for levels of infectious CHIKV at the times indicated by standard plaque assay on Vero cells, while cells were assayed for infectivity and induction of apoptosis by flow cytometry after staining with an anti-alphavirus monoclonal antibody and FITC-conjugated Annexin V/propidium iodide respectively. B. U937 cells were mock infected (M) or infected with ECSA CHIKV E1: A226 (EA), ECSA CHIKV E1: 226 V (EV) or Ross strain (RO) at m.o.i. of 1 and assayed for infectivity and induction of apoptosis by flow cytometry on the days indicated. All experiments were undertaken independently in triplicate with duplicate analysis of virus titers. Error bars show S.D. (TIF) [file pone.0031102.s007.tif]

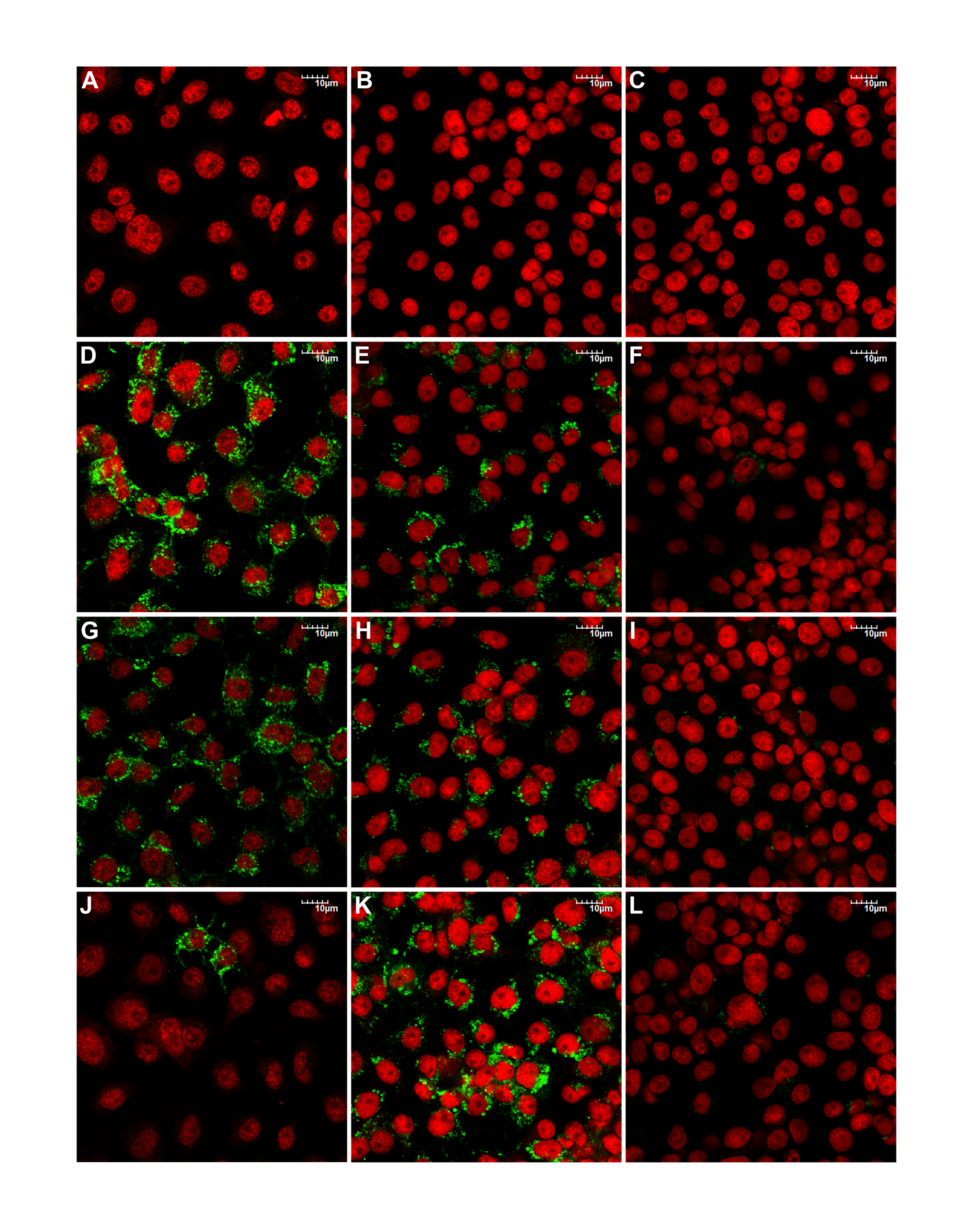

Supplement: Figure S8 — Analysis of CHIKV infected C6/36 cells. C6/36 cells were mock infected (A to C) or infected with CHIKV E1: 226 V (D to F), CHIKV E1: A226 (G to I) or Ross strain (J to L) at m.o.i. of 1 and subsequently stained with a mouse anti-alphavirus monoclonal antibody followed by a FITC conjugated goat anti-mouse IgG polyclonal antibody (green). Nuclei of cells were stained with TO-PRO-3 iodide (red) on days 1 (A, D, G and J), 3 (B, E, H and K) and 5 (C, F, I and L) p.i. Non-contrast adjusted merged images are shown. (TIF) [file pone.0031102.s008.tif]

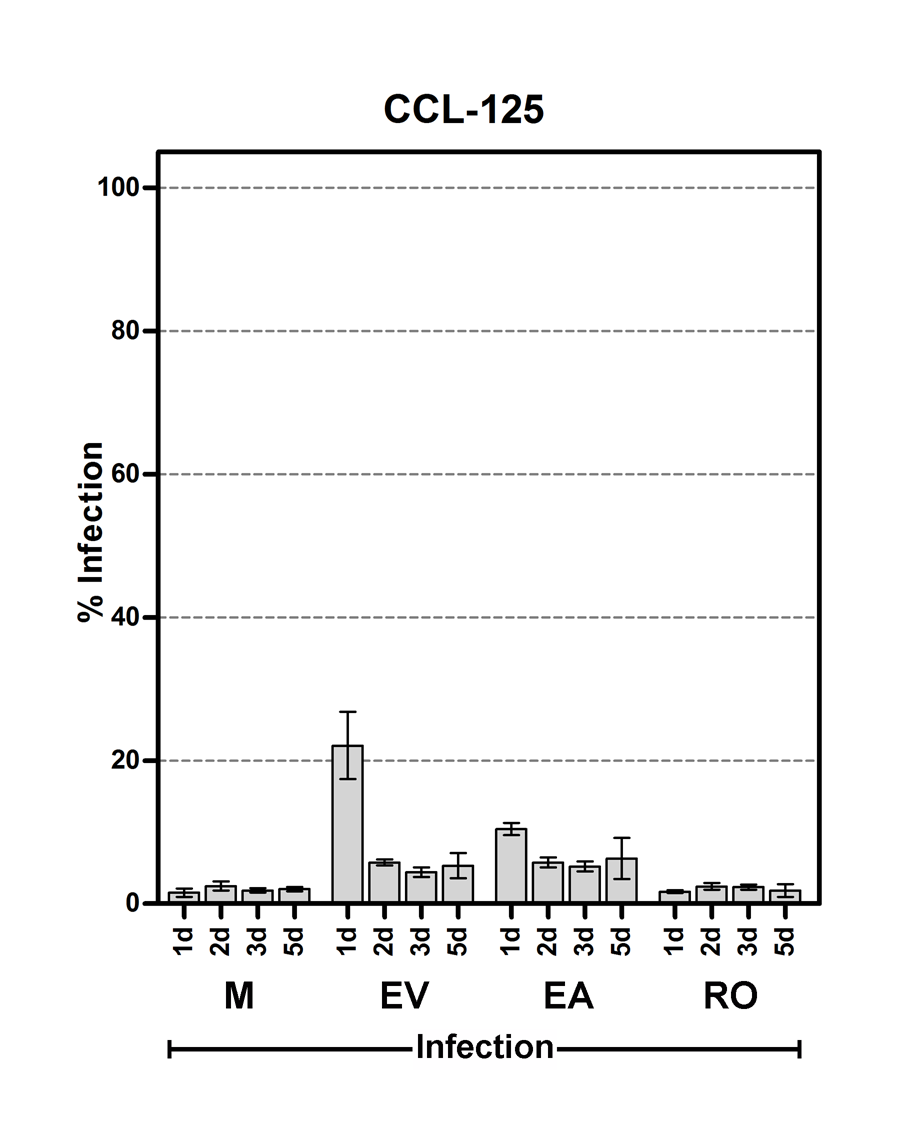

Supplement: Figure S9 — CHIKV infection of CCL-125 Aedes aegypti cells. A. CCL-125 cells were mock infected (M) or infected with CHIKV ECSA E1: 226 V (EV) at m.o.i. of 1. Infected cells and culture medium were collected daily and assayed for infectivity by flow cytometry after staining with an anti-alphavirus monoclonal antibody. (TIF) [file pone.0031102.s009.tif]
